# Supplementary material for: Nutrient-induced acidification modulates soil biodiversity-function relationships
Source: Nat Commun. 2024 Apr 3;15:2858. doi: 10.1038/s41467-024-47323-3 (PMC10991381; doi:10.1038/s41467-024-47323-3)
Supplement: Supplementary file 1 — Supplementary Information [file 41467_2024_47323_MOESM1_ESM.pdf]

Supplementary information

Nutrient-induced acidification modulates soil biodiversity-function relationships

Zhengkun Hu[1,2], Manuel Delgado-Baquerizo[3], Nicolas Fanin[4], Xiaoyun Chen[1], Yan Zhou[1], Guozhen Du[5], Feng Hu[1], Lin Jiang[6], Shuijin Hu[7], Manqiang Liu[2*]

Affiliations of authors:

[1] College of Resources and Environmental Sciences, Nanjing Agricultural University, Nanjing 210095, China

[2] Centre for Grassland Microbiome, State Key Laboratory of Herbage Improvement and Grassland Agro-Ecosystems, College of Pastoral Agriculture Science and Technology, Lanzhou University, Lanzhou 730020, China

[3] Laboratorio de Biodiversidad y Funcionamiento Ecosistémico. Instituto de Recursos Naturales y Agrobiología de Sevilla (IRNAS), CSIC, Av. Reina Mercedes 10, E-41012, Sevilla, Spain

[4] INRAE, Bordeaux Sciences Agro, UMR 1391 ISPA, Villenave-d'Ornon, France

[5] College of Ecology, Lanzhou University, Lanzhou, 730000, China

[6] School of Biological Sciences, Georgia Institute of Technology, Atlanta, USA

[7] Department of Entomology & Plant Pathology, North Carolina State University, Raleigh, NC, USA

*Corresponding author: Manqiang Liu; Email address: liumq@njau.edu.cn

Tel: +86 25 84395104; Fax: +86 25 84395210

This pdf file includes:

Supplementary Fig. 1 to 16

Supplementary Table 1

Supplementary References

Supplementary code

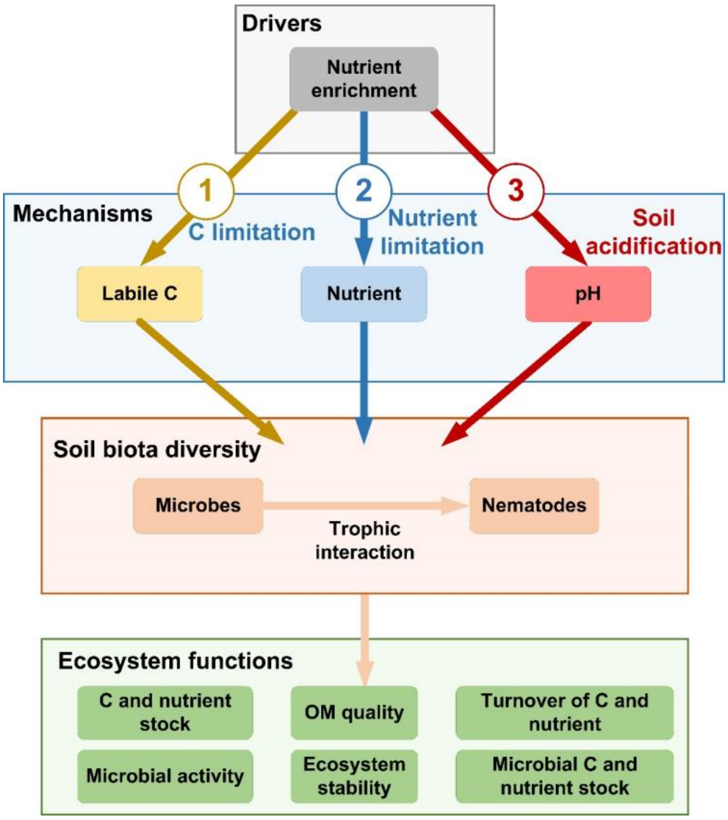

Supplementary Fig. 1 Schematic diagram of the effects of nutrient enrichment on soil biota diversity and ecosystem functions when considering each of three mechanisms. Mechanism 1 (yellow arrows, ‘C limitation’ hypothesis); Mechanism 2 (blue arrows, ‘nutrient limitation’ hypothesis); Mechanisms 3 (red arrows, ‘soil acidification’ hypothesis).

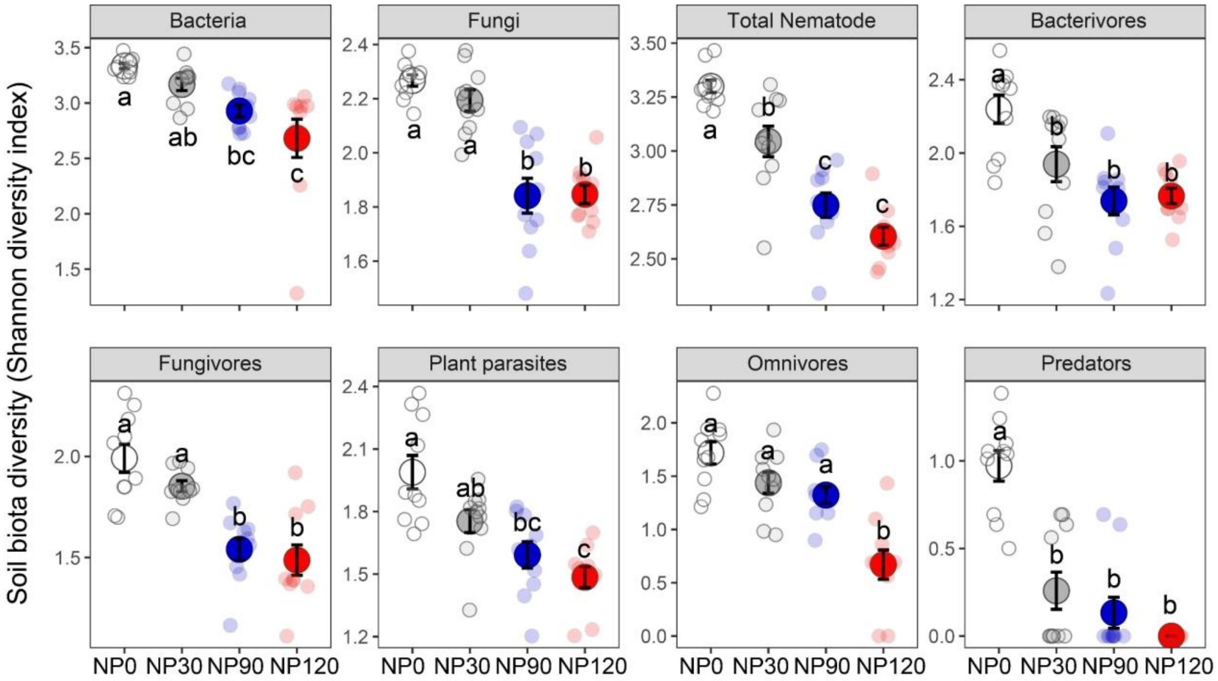

Supplementary Fig. 2 Effects of nutrient enrichment on the diversity of soil organisms. Soil biota diversity was calculated using Shannon diversity index. Dots with bars indicate Mean ± standard error (SE) (n = 10 independent soil samples per treatment), and jittered points represent biologically independent samples for each treatment. Data were analyzed using one-way ANOVA. Based on two-sided tests for multiple comparisons (Fisher's least significant difference), means with different lowercase letters indicate significant difference among treatments ($P < 0.05$). Source data are provided as a Source Data file.

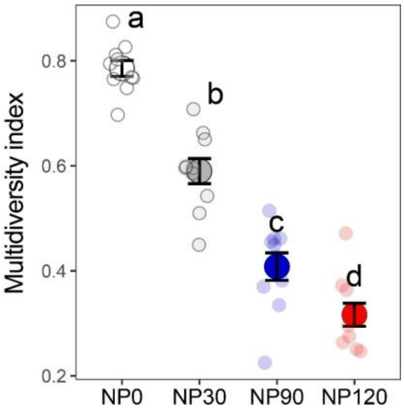

Supplementary Fig. 3 Effects of nutrient enrichment on soil multidiversity index. Dots with bars indicate Mean ± standard error (SE) (n = 10 independent soil samples per treatment), and jittered points represent biologically independent samples for each treatment. Data were analyzed using one-way ANOVA. Based on two-sided tests for multiple comparisons (Fisher's least significant difference), means with different lowercase letters indicate significant difference among treatments ($P < 0.05$). Source data are provided as a Source Data file.

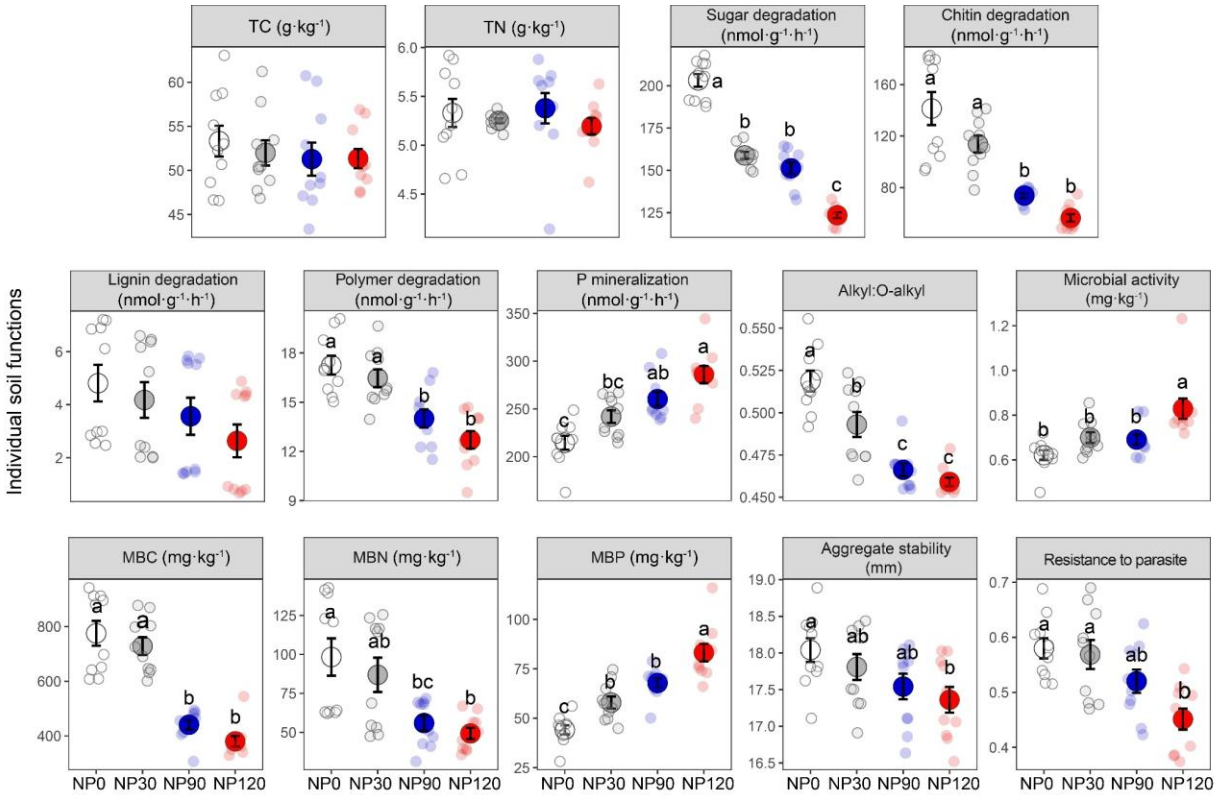

Supplementary Fig. 4 Effects of nutrient enrichment on soil individual functions. Dots with bars indicate Mean ± standard error (SE) (n = 10 independent soil samples per treatment), and jittered points represent biologically independent samples for each treatment. Data were analyzed using one-way ANOVA. Based on two-sided tests for multiple comparisons (Fisher's least significant difference), means with different lowercase letters indicate significant difference among treatments ($P < 0.05$). TC, total soil carbon; TN, total soil nitrogen; MBC, microbial biomass carbon; MBN, microbial biomass nitrogen; MBP, microbial biomass phosphorus. Source data are provided as a Source Data file.

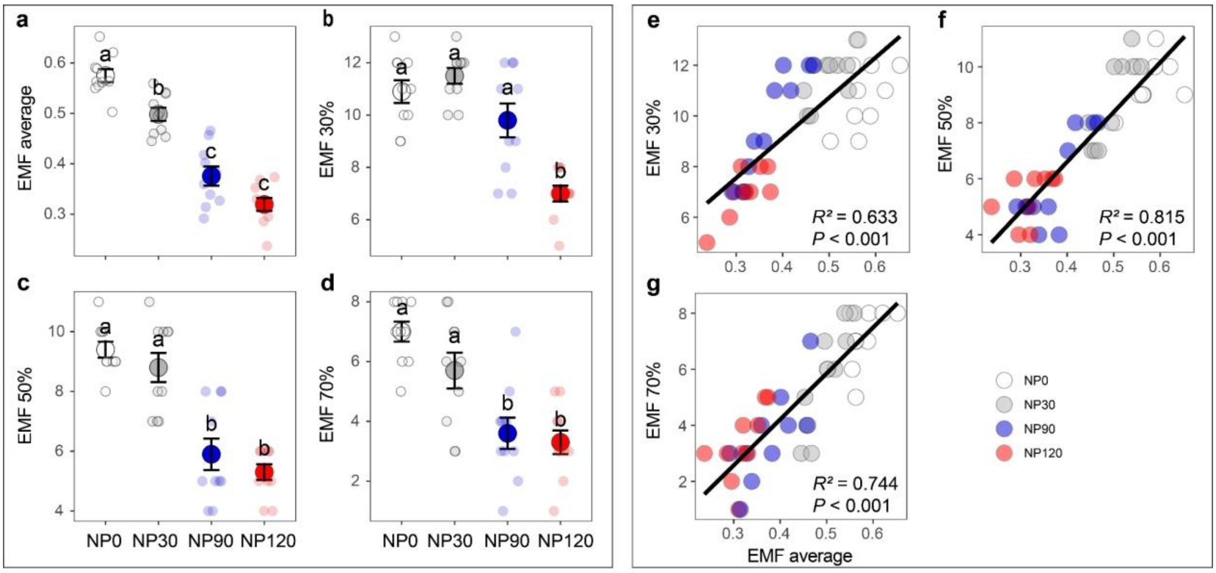

Supplementary Fig. 5 Effects of nutrient enrichment on the value of multifunctionality indices and the relationships among EMF indices. a-d Effects of nutrient enrichment on the value of multifunctionality indices as calculated by averaging approach and threshold approach at 30% (EMF 30%), 50% (EMF 50%) and 70% (EMF 70%) thresholds. Dots with bars indicate Mean ± standard error (SE) (n = 10 independent soil samples per treatment), and jittered points represent biologically independent samples for each treatment. Data were analyzed using one-way ANOVA. Based on two-sided tests for multiple comparisons (Fisher's least significant difference), means with different lowercase letters indicate significant difference among treatments (P < 0.05). **e-g** The relationships between average EMF and EMF 30%, EMF 50% and EMF 70% as influenced by nutrient enrichment. Linear regression model with two-sided test was used for the statistical analysis, and multiple R-squared was used. Relationships are denoted with solid lines and fit statistics (R^2 and P values). The solid line represents the significant linear regression (P < 0.05). n = 40 independent soil samples for regression model. Source data are provided as a Source Data file.

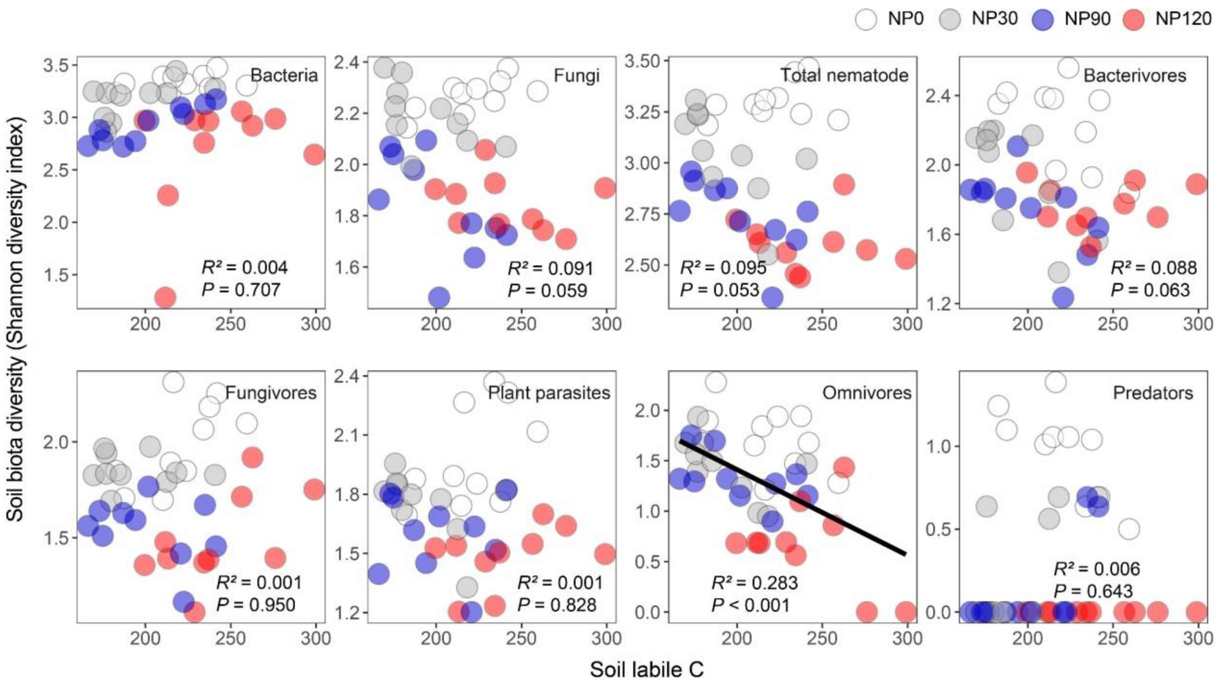

Supplementary Fig. 6 Relationships between soil labile C and the diversity of soil biota as influenced by nutrient enrichment. Linear regression model with two-sided test was used for the statistical analysis, and multiple R-squared was used. Relationships are denoted with solid lines and fit statistics (R^2 and P values). The solid line represents the significant linear regression ($P < 0.05$). $n = 40$ independent soil samples for regression model. Source data are provided as a Source Data file.

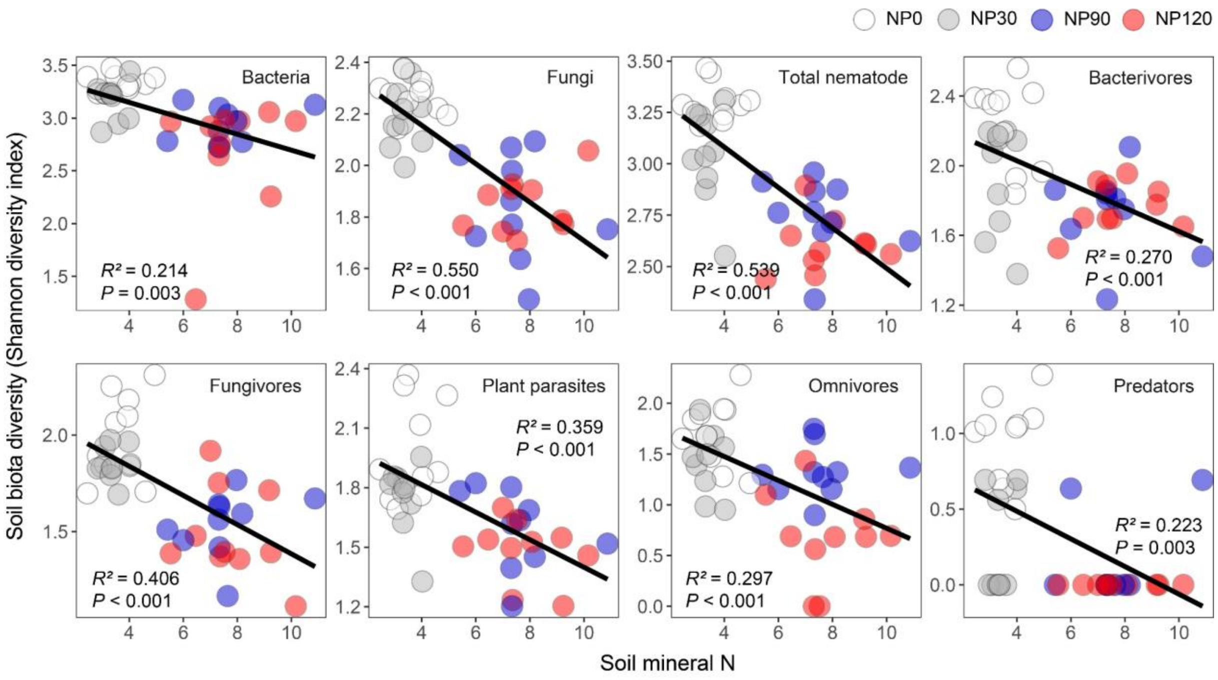

Supplementary Fig. 7 Relationships between soil mineral N and the diversity of soil biota as influenced by nutrient enrichment. Linear regression model with two-sided test was used for the statistical analysis, and multiple R-squared was used. Relationships are denoted with solid lines and fit statistics (R^2 and P values). The solid line represents the significant linear regression (P < 0.05). n = 40 independent soil samples for regression model. Source data are provided as a Source Data file.

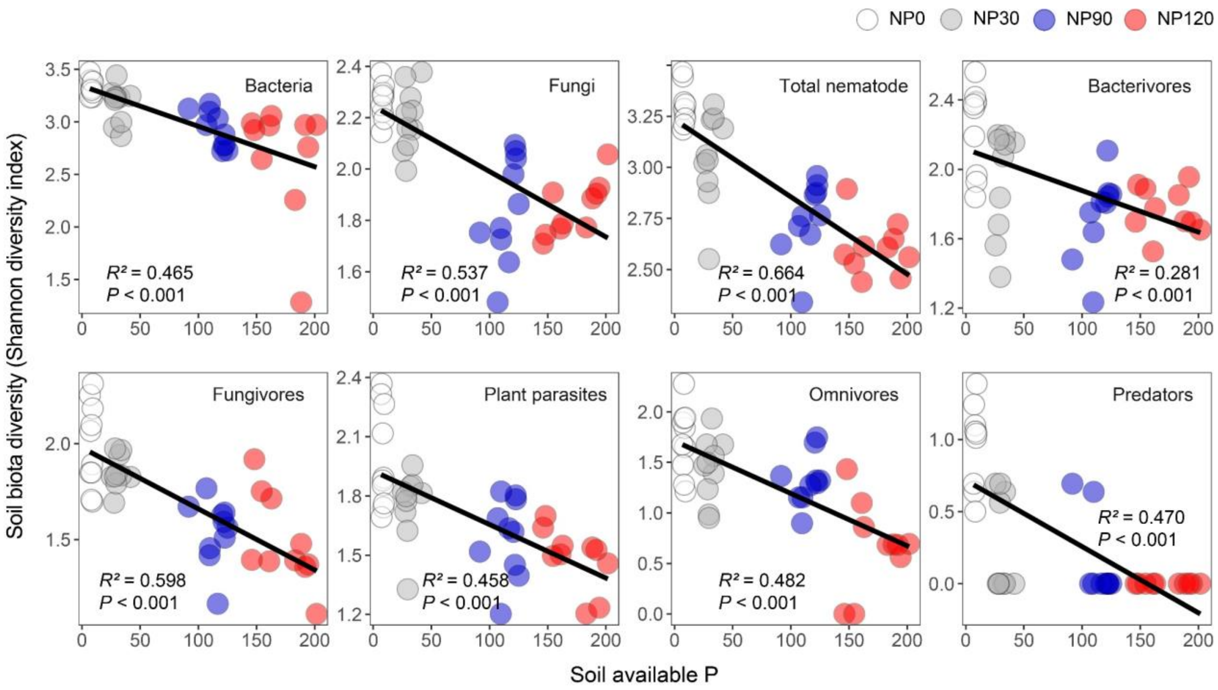

Supplementary Fig. 8 Relationships between soil available P and the diversity of soil biota as influenced by nutrient enrichment. Linear regression model with two-sided test was used for the statistical analysis, and multiple R-squared was used. Relationships are denoted with solid lines and fit statistics (R^2 and P values). The solid line represents the significant linear regression ($P < 0.05$). n = 40 independent soil samples for regression model. Source data are provided as a Source Data file.

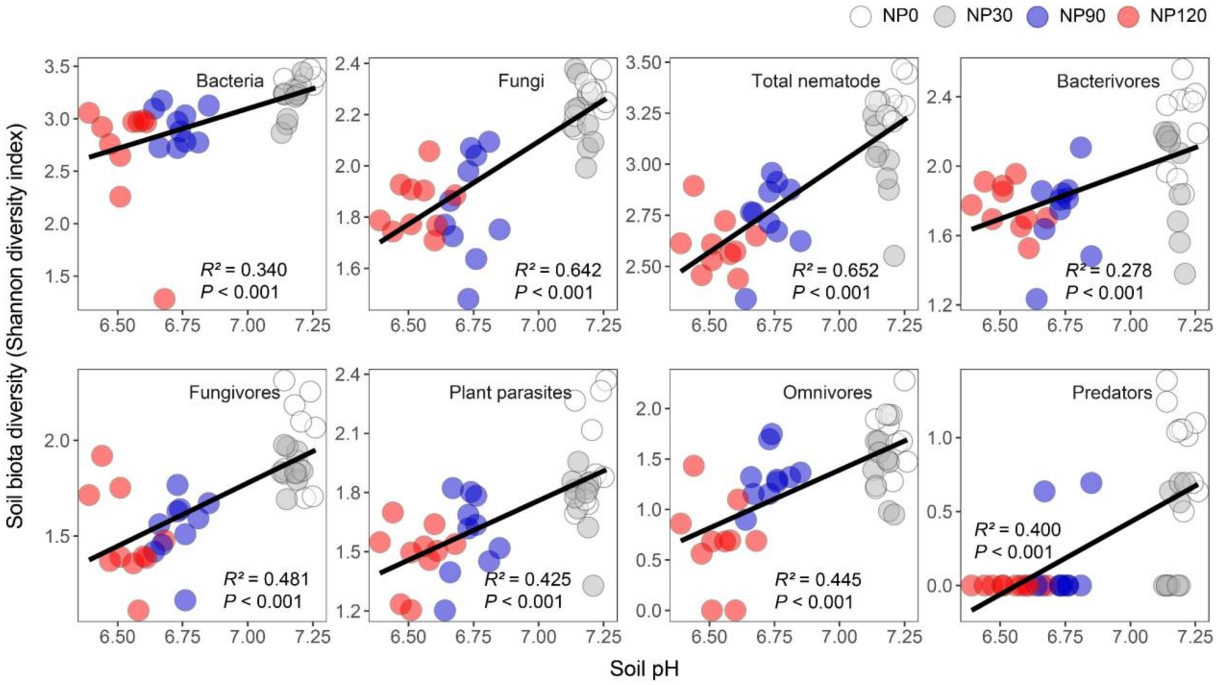

Supplementary Fig. 9 Relationships between soil pH and the diversity of soil biota as influenced by nutrient enrichment. Linear regression model with two-sided test was used for the statistical analysis, and multiple R-squared was used. Relationships are denoted with solid lines and fit statistics (R^2 and P values). The solid line represents the significant linear regression ($P < 0.05$). n = 40 independent soil samples for regression model. Source data are provided as a Source Data file.

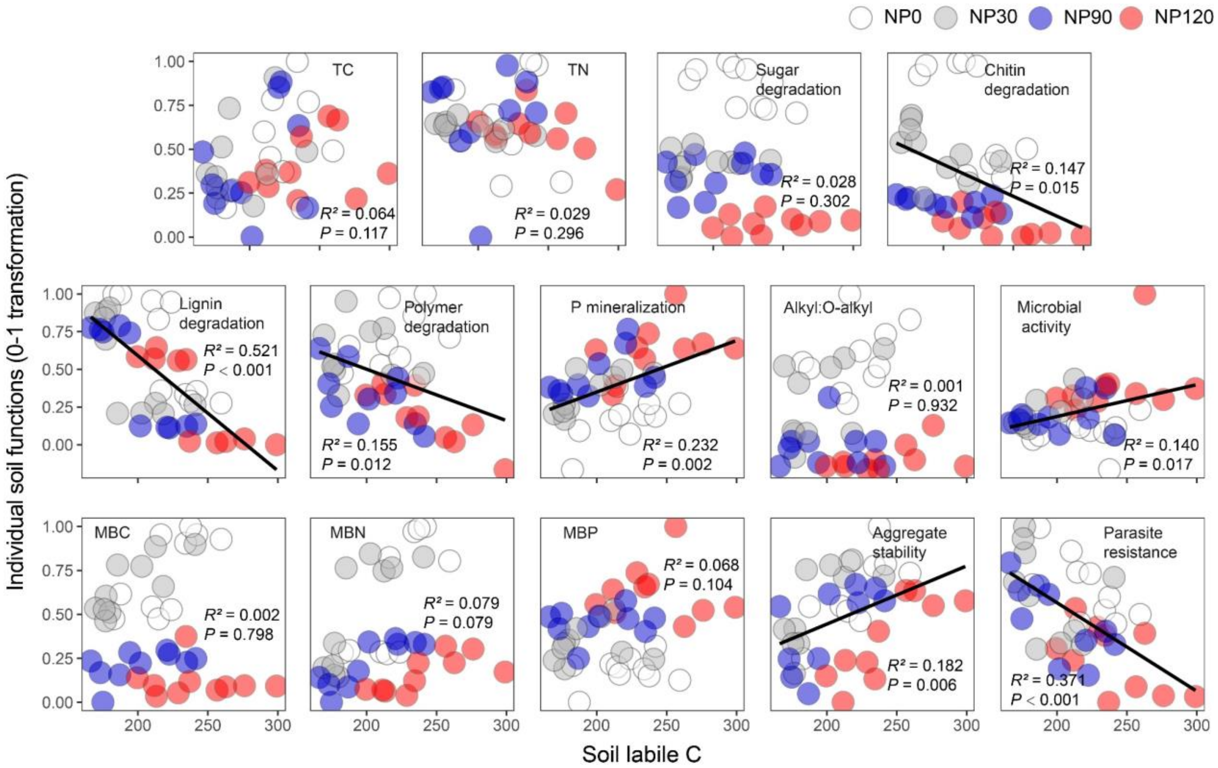

Supplementary Fig. 10 Relationships between soil labile C and individual soil functions as influenced by nutrient enrichment. Linear regression model with two-sided test was used for the statistical analysis, and multiple R-squared was used. Relationships are denoted with solid lines and fit statistics (R^2 and P values). The solid line represents the significant linear regression (P < 0.05). n = 40 independent soil samples for regression model. TC, total soil carbon; TN, total soil nitrogen; MBC, microbial biomass carbon; MBN, microbial biomass nitrogen; MBP, microbial biomass phosphorus. Source data are provided as a Source Data file.

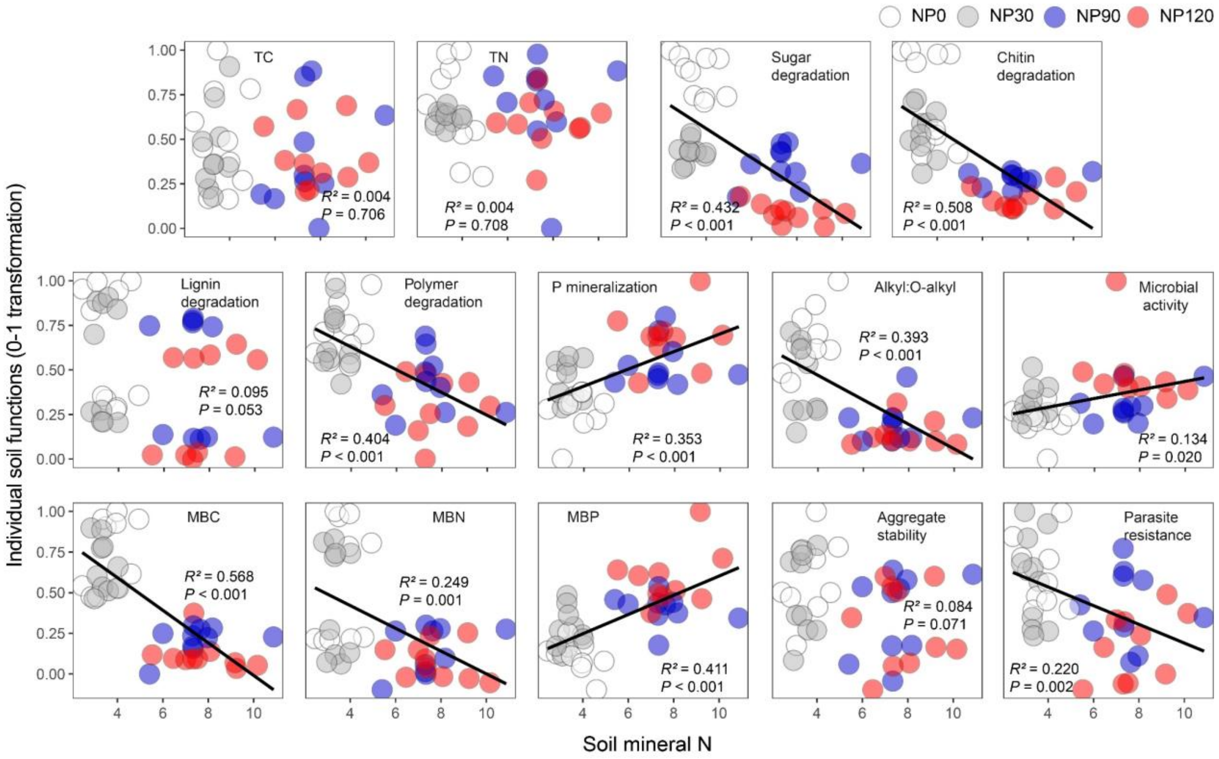

Supplementary Fig. 11 Relationships between soil mineral N and individual soil functions as influenced by nutrient enrichment. Linear regression model with two-sided test was used for the statistical analysis, and multiple R-squared was used. Relationships are denoted with solid lines and fit statistics (R^2 and P values). The solid line represents the significant linear regression ($P < 0.05$). n = 40 independent soil samples for regression model. TC, total soil carbon; TN, total soil nitrogen; MBC, microbial biomass carbon; MBN, microbial biomass nitrogen; MBP, microbial biomass phosphorus. Source data are provided as a Source Data file.

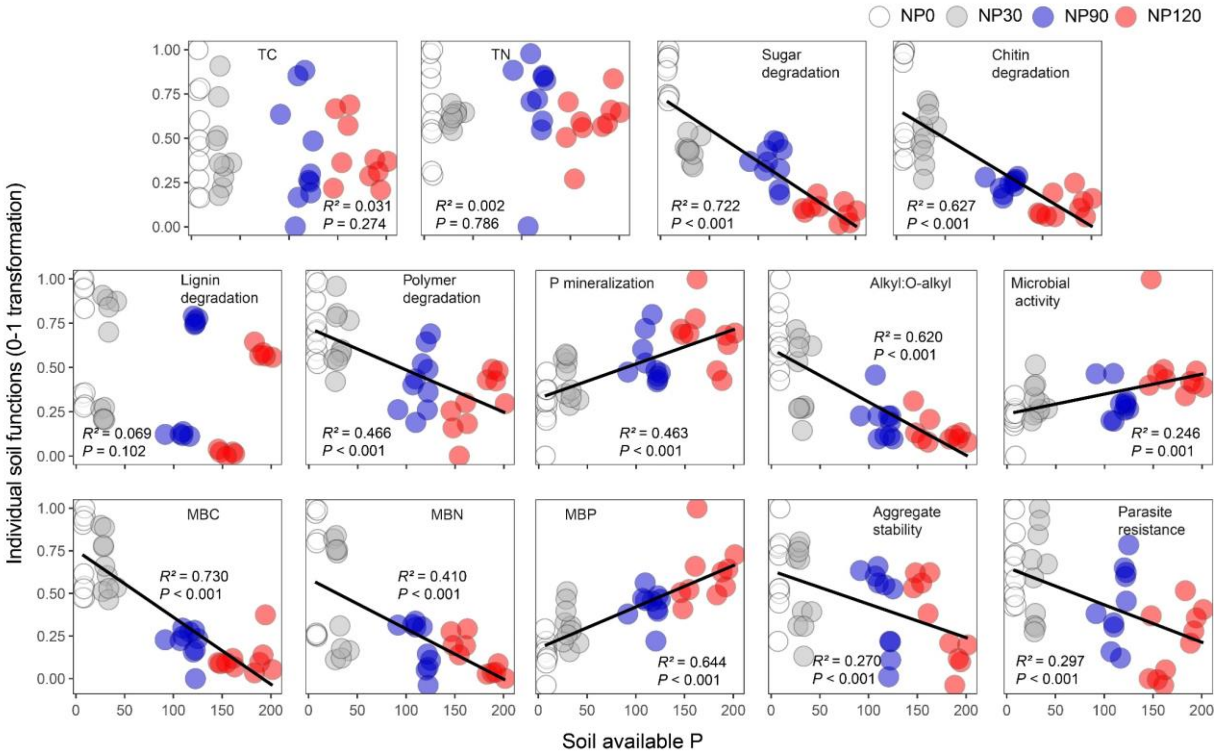

Supplementary Fig. 12 Relationships between soil available P and individual soil functions as influenced by nutrient enrichment. Linear regression model with two-sided test was used for the statistical analysis, and multiple R-squared was used. Relationships are denoted with solid lines and fit statistics (R^2 and P values). The solid line represents the significant linear regression (P < 0.05). n = 40 independent soil samples for regression model. TC, total soil carbon; TN, total soil nitrogen; MBC, microbial biomass carbon; MBN, microbial biomass nitrogen; MBP, microbial biomass phosphorus. Source data are provided as a Source Data file.

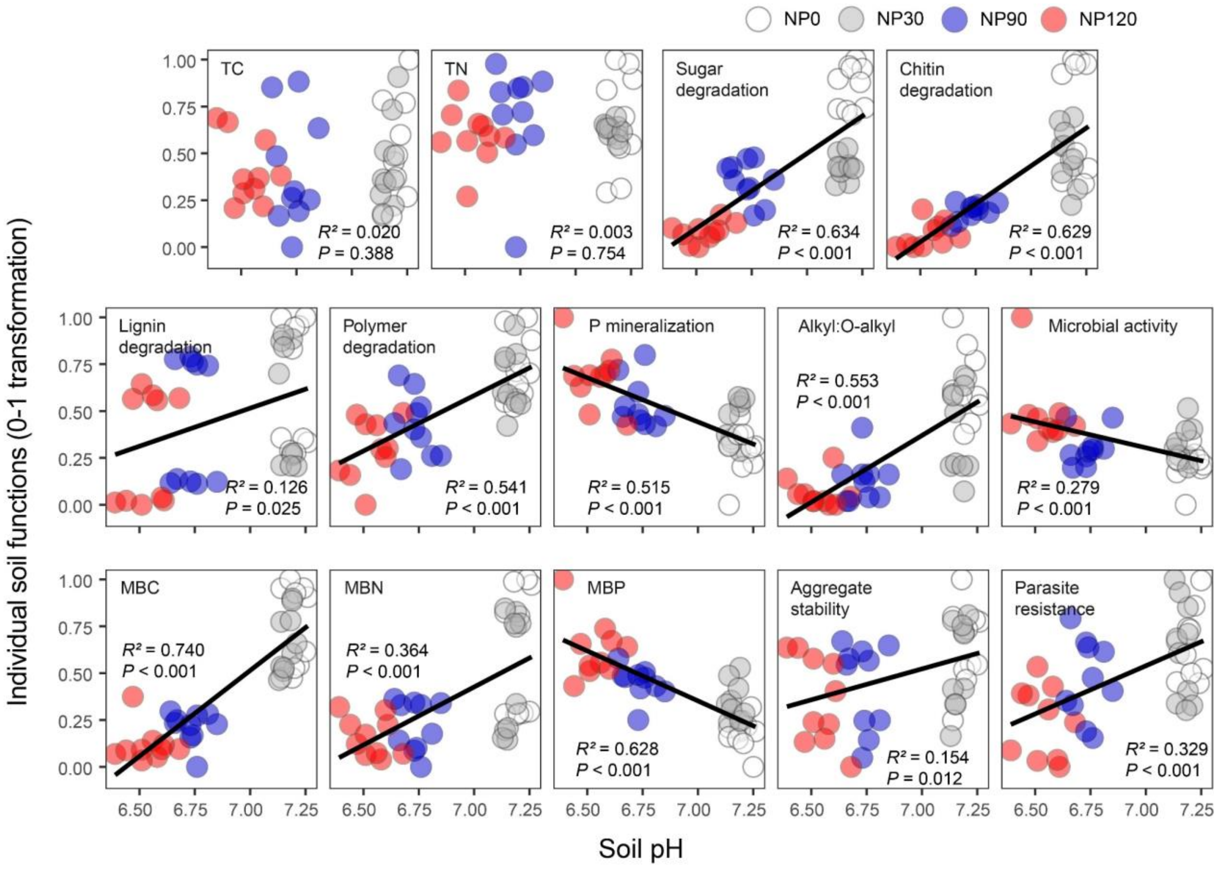

Supplementary Fig. 13 Relationships between soil pH and individual soil functions as influenced by nutrient enrichment. Linear regression model with two-sided test was used for the statistical analysis, and multiple R-squared was used. Relationships are denoted with solid lines and fit statistics (R^2 and P values). The solid line represents the significant linear regression ($P < 0.05$). n = 40 independent soil samples for regression model. TC, total soil carbon; TN, total soil nitrogen; MBC, microbial biomass carbon; MBN, microbial biomass nitrogen; MBP, microbial biomass phosphorus. Source data are provided as a Source Data file.

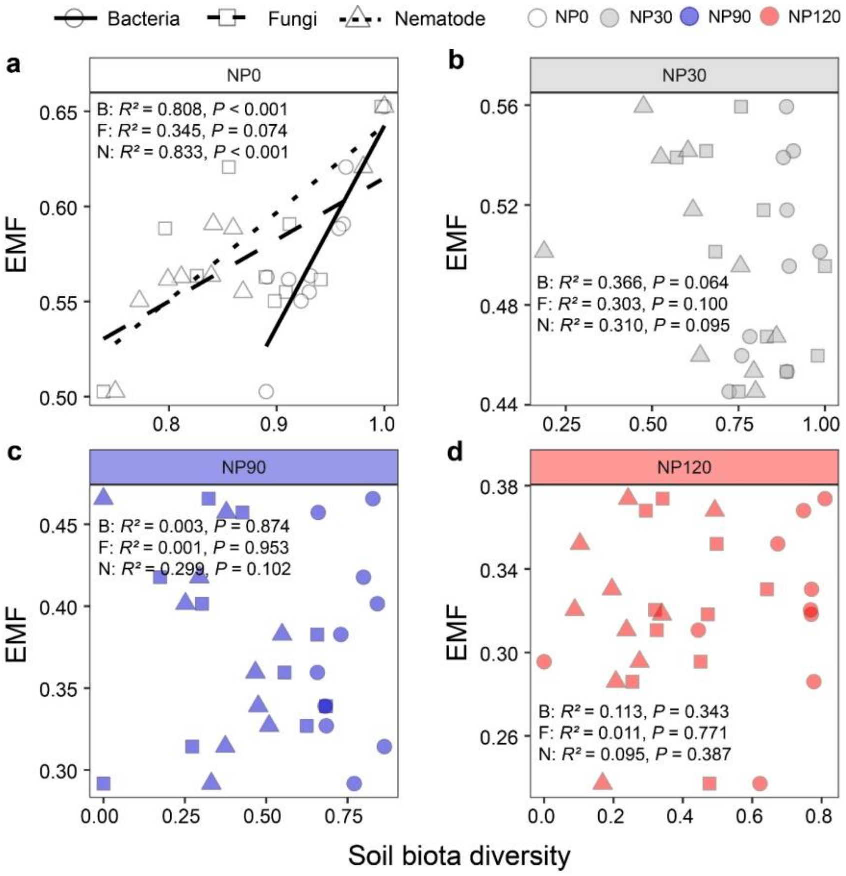

Supplementary Fig. 14 Nutrient enrichment weakened the relationship between the diversity of single soil biota group and EMF at all three nutrient addition levels. The fitted linear relationships between soil biodiversity and EMF under **a** NP0, **b** NP30, **c** NP90, **d** NP120. Linear regression model with two-sided test was used for the statistical analysis, and multiple R-squared was used. Relationships are denoted with solid lines and fit statistics (R^2 and P values). The solid line represents the significant linear regression ($P < 0.05$). $n = 10$ independent soil samples for regression model. B: bacteria; F: Fungi; N: nematode. Source data are provided as a Source Data file.

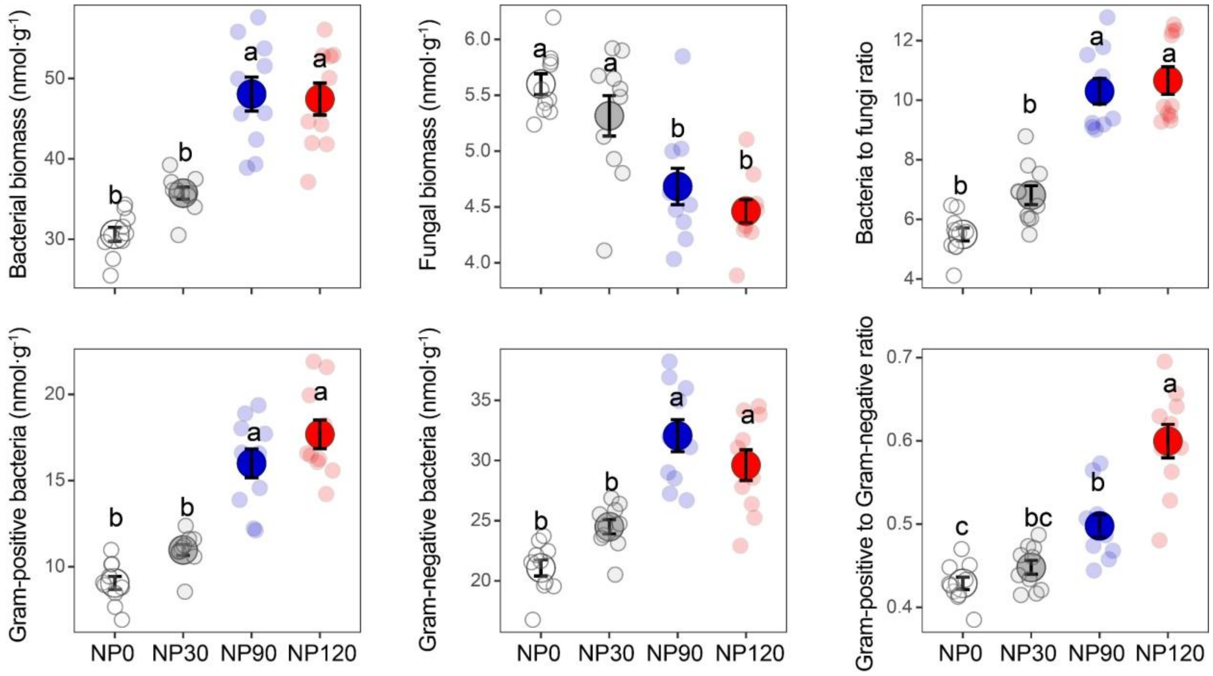

Supplementary Fig. 15 Effects of nutrient enrichment on microbial community structure. Microbial biomass was measured by phospholipid fatty acid analysis[1]. Briefly, lipid was extracted from 8.0 g freeze-dried soil with a chloroform–methanol–citrated buffer mixture (25ml at a 1:2:0.8 v/v), and the phospholipids were transesterified to a mild alkaline methanolysis. The resulting fatty acid methyl esters were extracted in hexane and dried under N_2 and were then dissolved in hexane and analyzed in an Agilent 6850 series gas chromatograph with MIDI peak identification software (v.4.5; MIDI Inc., Newark, DE, USA). The fatty acids i14:0, i15:0, a15:0, i16:0, 16:1ω7c, i17:0, a17:0, 17:0cy, 18:1ω9, 18:1ω7c, and 19:0cy were chosen as bacterial markers; 18:1ω9c and 18:2ω6.9c were used as fungal markers. Dots with bars indicate Mean ± standard error (SE) (n = 10 independent soil samples per treatment), and jittered points represent biologically independent samples for each treatment. Data were analyzed using one-way ANOVA. Based on two-sided tests for multiple comparisons (Fisher's least significant difference), means with different lowercase letters indicate significant difference among treatments ($P < 0.05$). Source data are provided as a Source Data file.

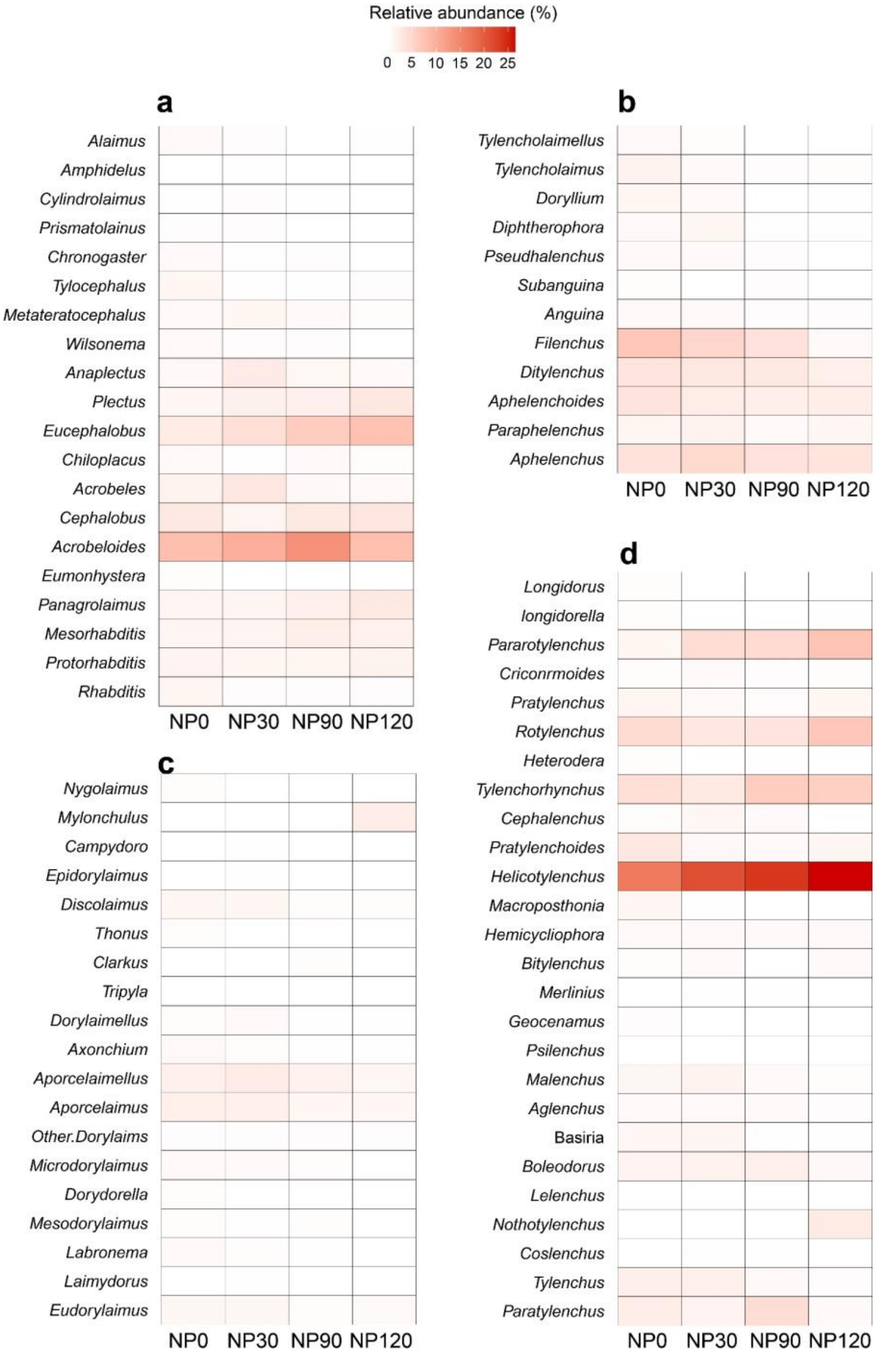

Supplementary Fig. 16 Effects of nutrient enrichment on the relative abundance of nematode genera. a Relative abundance of bacterivores. **b** relative abundance of fungivores. **c** Relative abundance of omnivores and predators. **d** Relative abundance of herbivores. n = 10 independent soil samples for regression model. Source data are provided as a Source Data file.

Supplementary Table 1 The individual ecosystem functions used for calculating ecosystem multifunctionality in the field and microcosm experiment.

Ecosystem functions	Units	Description
Total soil C	g/kg	SOC usually interacts with many biotic and abiotic components of ecosystems and plays important role in regulating climate
Total soil N	g/kg	Build-up of nutrient pools that most frequently limit the primary production in diverse ecosystems
Sugar degradation	nmol/g h	Indicator of the activity of the microbial community and its capacity to drive organic matter decomposition in terrestrial ecosystem[2,3]
Chitin degradation	nmol/g h	
Lignin degradation	nmol/g h	
Polymer degradation	mg/kg	
P mineralization	nmol/g h	
alkyl:o-alkyl	unitless	Indicator of OM quality and microbial processing, with higher ratios indicating losses of more labile C relative to more stable C compounds[4,5]
Microbial activity	mg C/kg	A measure of the potential activity of the component of the microbial community that is responsible for driving decomposition and related processes[6,7]
MBC	mg/kg	Early indicator of changes in soil chemical and physical properties; the most labile C, N, and P pools in soil affecting nutrient availability and plant productivity[3,8]
MBN	mg/kg	
MBP	mg/kg	
Aggregate stability	mm	Indicator of soil structure influencing storage of water, aeration, erosion, biological activity and the growth of crops[9,10]
Resistance to parasites	unitless	Reduced relative abundance of plant parasitic nematode; Indicator of the capacity of soil systems to resistant plant parasites[11–13]

Supplementary Code. Code for all analyses

1. code for analyses in the main text

#Figure1 Effects of nutrient enrichment on soil physicochemical properties

```
#Anova
library(psych)
library(agricolae)
setwd("D:/Submission/8-Fertilization-EMF/NC-revision/Final/data")
EMF<-read.csv(file="Rawdata.csv")
myaov=aov(LabileC~Treatment,data=EMF) #"LabileC, MineralN, AvailableP, pH"
summary(myaov)
LSD.test(myaov,"Treatment", p.adj="bonferroni", console=T)

#Figure
library(Rmisc)
library(ggplot2)
library (reshape2)
setwd("D:/Submission/8-Fertilization-EMF/NC-revision/Final/data")
EMF<-read.csv(file="Rawdata.csv")
EMF<-melt(EMF,id.vars=1:6, measure.vars = c(9,7,8,10),
          variable.name = "Function", value.name = "value")
myhisto<-summarySE(EMF, measurevar="value",groupvars=c("Function","Treatment"))
myhisto$Treatment<-factor(myhisto$Treatment,levels=c("NP0","NP30","NP90","NP120"))
ggplot(myhisto, aes(x=Treatment, y=value,fill=Treatment,colour=Treatment))+
  geom_point(position=position_dodge(0.8),stat="identity",size=6, shape=21)+
  geom_errorbar(aes(ymin=value-
se,ymax=value+se),width=0.2,colour="black",position=position_dodge(0.8),size=0.8)+
  geom_jitter(data=EMF,aes(fill=Treatment),position=position_jitter(0.2),shape=21, size=3, alpha=0.2)+
  facet_wrap(~Function,scales="free",nrow = 2)+     #,nrow = 4
  theme_bw()+
  theme(panel.background = element_blank(),
        panel.grid.major = element_blank(),
        panel.grid.minor = element_blank(),plot.background = element_blank(),
        axis.text.x = element_text(size=12,colour="black", angle = 0, hjust = 0.5),
        axis.text.y = element_text(size = 10)) +
  scale_colour_manual(values=c("grey30","grey30","navy","tomato4"))+
  scale_fill_manual(values=c("white","grey70","blue3","red"))
```

####Figure 2 Relationships between soil physicochemical properties and soil multidiversity as influenced by nutrient enrichment

```
#Regression
setwd("D:/Submission/8-Fertilization-EMF/NC-revision/Final/data")
EMF<-read.csv(file="Rawdata.csv")
x<-EMF[,c("LabileC")] #"LabileC, MineralN, AvailableP, pH"
y<-EMF[,c("Multidiversity")]
cor.test(x,y,method = "pearson")
test=lm(y~x)
test
summary(test)

#Figure
library(Rmisc)
library(ggplot2)
library (reshape2)
setwd("D:/Submission/8-Fertilization-EMF/NC-revision/Final/data")
EMF<-read.csv(file="Rawdata.csv")
```

```r
EMF<-melt(EMF,id.vars=c(1:6,23,55,48), measure.vars = c(9,7,8,10),
                   variable.name = "Function", value.name = "value")
EMF$Treatment<-factor(EMF$Treatment,levels=c("NP0","NP30","NP90","NP120"))
p1<-ggplot(data=EMF,mapping=aes(x=value,y=Multidiversity,shape=Treatment,fill=Treatment,colour=Treatment))+ #PredatorToPreyB
   geom_point(size=6,alpha=0.5)+
   scale_shape_manual(values = c(21,21,21,21))+
   scale_colour_manual(values =c("grey30","grey30","navy","tomato4"))+
   scale_fill_manual(values = c("white","grey70","blue3","red"))+
   geom_smooth(aes(group=All,linetype=All), color="black",method="lm", size=1.5, se=F)+
   scale_linetype_manual(values=c(1))+
   facet_wrap(~Function,scales="free",nrow = 2)+
   theme_bw()+
   theme(axis.text.x = element_text(size=12, angle = 0, hjust = 0.5),
            axis.text.y = element_text(size = 12),
            axis.title=element_text(size=14,face="bold"),
            axis.ticks.length = unit(0.1,"cm"),
            panel.grid = element_blank(),
            panel.background = element_blank(),
            panel.grid.major = element_blank(),
            panel.grid.minor = element_blank(),plot.background = element_blank())
p1


####Figure 3 Relationships between soil prey and predator diversity as influenced by nutrient enrichment
#Regression
setwd("D:/Submission/8-Fertilization-EMF/NC-revision/Final/data")
EMF<-read.csv(file="Rawdata.csv")
x<-EMF[,c("Microbialdiversity")] #"Preydiversity"
y<-EMF[,c("Microbivorediversity")]    #"Predatordiversity"
cor.test(x,y,method = "pearson")
test=lm(y~x)
test
summary(test)

#Figures
library(Rmisc)
library(ggplot2)
library (reshape2)
setwd("D:/Submission/8-Fertilization-EMF/NC-revision/Final/data")
EMF<-read.csv(file="Rawdata.csv")
EMF$Treatment<-factor(EMF$Treatment,levels=c("NP0","NP30","NP90","NP120"))
p1<-ggplot(data=EMF,mapping=aes(x=Microbialdiversity,y=Microbivorediversity,shape=Treatment,fill=Treatment,colour=Treatment))+ #PredatorToPreyB
   geom_point(size=6,alpha=0.5)+
   scale_shape_manual(values = c(21,21,21,21))+
   scale_colour_manual(values =c("grey30","grey30","navy","tomato4"))+
   scale_fill_manual(values = c("white","grey70","blue3","red"))+
   geom_smooth(aes(group=All,linetype=All), color="black",method="lm", size=1.5, se=F)+
   scale_linetype_manual(values=c(1))+

   theme_bw()+
   theme(axis.text.x = element_text(size=12, angle = 0, hjust = 0.5),
            axis.text.y = element_text(size = 12),
            axis.title=element_text(size=14,face="bold"),
```

```
             axis.ticks.length = unit(0.1,"cm"),
             panel.grid = element_blank(),
             panel.background = element_blank(),
             panel.grid.major = element_blank(),
             panel.grid.minor = element_blank(),plot.background = element_blank())
p1


p2<-
ggplot(data=EMF,mapping=aes(x=Preydiversity,y=Predatordiversity,shape=Treatment,fill=Treatment,colou
r=Treatment))+ #PredatorToPreyB
   geom_point(size=6,alpha=0.5)+
   scale_shape_manual(values = c(21,21,21,21))+
   scale_colour_manual(values =c("grey30","grey30","navy","tomato4"))+
   scale_fill_manual(values = c("white","grey70","blue3","red"))+
   geom_smooth(aes(group=All,linetype=All), color="black",method="lm", size=1.5, se=F)+
   scale_linetype_manual(values=c(1))+

   theme_bw()+
   theme(axis.text.x = element_text(size=12, angle = 0, hjust = 0.5),
             axis.text.y = element_text(size = 12),
             axis.title=element_text(size=14,face="bold"),
             axis.ticks.length = unit(0.1,"cm"),
             panel.grid = element_blank(),
             panel.background = element_blank(),
             panel.grid.major = element_blank(),
             panel.grid.minor = element_blank(),plot.background = element_blank())
p2

library(cowplot)
p3 <- plot_grid( p1, p2, nrow = 1, align = "v",labels = letters[1:2])
p3
```

####Figure 4 Relationships between soil physicochemical properties and soil multidiversity as influenced by nutrient enrichment

```
#Regression
setwd("D:/Submission/8-Fertilization-EMF/NC-revision/Final/data")
EMF<-read.csv(file="Rawdata.csv")
x<-EMF[,c("AvailableP")] #"LabileC, MineralN, AvailableP, pH"
y<-EMF[,c("Multifunction")]
cor.test(x,y,method = "pearson")
test=lm(y~x)
test
summary(test)

#Figure
library(Rmisc)
library(ggplot2)
library (reshape2)
setwd("D:/Submission/8-Fertilization-EMF/NC-revision/Final/data")
EMF<-read.csv(file="Rawdata.csv")
EMF<-melt(EMF,id.vars=c(1:6,23,44,48), measure.vars = c(9,7,8,10),
               variable.name = "Function", value.name = "value")
EMF$Treatment<-factor(EMF$Treatment,levels=c("NP0","NP30","NP90","NP120"))
p1<-
ggplot(data=EMF,mapping=aes(x=value,y=Multifunction,shape=Treatment,fill=Treatment,colour=Treatme
nt))+ #PredatorToPreyB
```

```
  geom_point(size=6,alpha=0.5)+
  scale_shape_manual(values = c(21,21,21,21))+
  scale_colour_manual(values =c("grey30","grey30","navy","tomato4"))+
  scale_fill_manual(values = c("white","grey70","blue3","red"))+
  geom_smooth(aes(group=All,linetype=All), color="black",method="lm", size=1.5, se=F)+
  scale_linetype_manual(values=c(1))+
  facet_wrap(~Function,scales="free",nrow = 2)+
  theme_bw()+
  theme(axis.text.x = element_text(size=12, angle = 0, hjust = 0.5),
        axis.text.y = element_text(size = 12),
        axis.title=element_text(size=14,face="bold"),
        axis.ticks.length = unit(0.1,"cm"),
        panel.grid = element_blank(),
        panel.background = element_blank(),
        panel.grid.major = element_blank(),
        panel.grid.minor = element_blank(),plot.background = element_blank())
p1
```

####Figure 5 Relationships between soil biodiversity and soil functions or ecosystem multifunctionality

```
#correlation analysis
#Figure5a
library(corrplot)
setwd("D:/Submission/8-Fertilization-EMF/NC-revision/Final/data")
EMF<-read.csv(file="Rawdata.csv")
x<-EMF[,c(54:61)]
y<-EMF[,c(62:75)]
matrix<-cor(y,x,use="everything",method= c("spearman")) #"kendall","spearman"
res1 <- cor.mtest(matrix, conf.level = .95)
corrplot(corr=matrix,method = "square",tl.col = "black"
         ,tl.pos = "lt")

#Relationship between soil biota diversity and EMF
setwd("D:/Submission/8-Fertilization-EMF/NC-revision/Final/data")
EMF<-read.csv(file="Rawdata.csv")
x<-EMF[,c("Bacterialdiversity1")]    #"Fungaldiversity1, Nematodediversity1, BFdiversity1,
FFdiversity1,PFdiversity1,OMdiversity1,PRdiversity1"
y<-EMF[,c("Multifunction")]
cor.test(x,y,method = "pearson")
test=lm(y~x)
test
summary(test)

#Figure5b
library(Rmisc)
library(ggplot2)
library (reshape2)
library(ggsci)
setwd("D:/Submission/8-Fertilization-EMF/NC-revision/Final/data")
EMF<-read.csv(file="Rawdata.csv")
Diversity<-melt(EMF,id.vars=c(1:6,44,23,48), measure.vars = c(54:61,23),
                variable.name = "Biota", value.name = "value")

p1<-ggplot(data=Diversity,mapping=aes(x=value,y=Multifunction,shape=All,fill=Biota),colour=Biota)+
  geom_smooth(aes(group=Biota,linetype=Biota,color=Biota), method="lm", size=1.5, se=F)+
  scale_linetype_manual(values=c(1,1,1,1,1,1,1,1,2))+
  theme_bw()+
```

```
  theme(axis.text.x = element_text(size=12,colour="black", angle = 0, hjust = 0.5),
        axis.text.y = element_text(size = 12,colour="black"),
        axis.title=element_text(size=14,face="bold"),
        axis.ticks.length = unit(0.1,"cm"),
        panel.spacing.x = unit(10, 'mm'),
        panel.grid = element_blank(),
        panel.background = element_blank(),
        panel.grid.major = element_blank(),
        panel.grid.minor = element_blank(),plot.background = element_blank())
plot(p1)
p2<-p1+scale_fill_npg()+scale_color_npg()
p2

#Relationships between multidiversity and multi-threshold EMF
#correlation
setwd("D:/Submission/8-Fertilization-EMF/NC-revision/Final/data")
EMF<-read.csv(file="Rawdata.csv")
x<-EMF[,c("Multidiversity")]
#"Fungaldiversity1, Nematodediversity1, BFdiversity1,
#FFdiversity1,PFdiversity1,OMdiversity1,PRdiversity1"
y<-EMF[,c("EMF3")]   #"EMF5,EMF7"
cor.test(x,y,method = "pearson")
test=lm(y~x)
test
summary(test)
#Figure5c
library(Rmisc)
library(ggplot2)
library (reshape2)
library(ggsci)
setwd("D:/Submission/8-Fertilization-EMF/NC-revision/Final/data")
EMF<-read.csv(file="Rawdata.csv")
Diversity<-melt(EMF,id.vars=c(1:6,23,48), measure.vars = 45:47,
                variable.name = "Biota", value.name = "value")

p3<-ggplot(data=Diversity,mapping=aes(x=Multidiversity,y=value,shape=All,fill=Biota),colour=Biota)+
  geom_smooth(aes(group=Biota,linetype=Biota,color=Biota), method="lm", size=1.5, se=F)+
  scale_linetype_manual(values=c(1,1,1,1,1,1,1,1,2))+
  theme_bw()+
  theme(axis.text.x = element_text(size=12,colour="black", angle = 0, hjust = 0.5),
        axis.text.y = element_text(size = 12,colour="black"),
        axis.title=element_text(size=14,face="bold"),
        axis.ticks.length = unit(0.1,"cm"),
        panel.spacing.x = unit(10, 'mm'),
        panel.grid = element_blank(),
        panel.background = element_blank(),
        panel.grid.major = element_blank(),
        panel.grid.minor = element_blank(),plot.background = element_blank())
plot(p3)
p4<-p3+scale_fill_npg()+scale_color_npg()
p4

####Figure 6 Nutrient enrichment weakened the diversity-function linkage at all three nutrient addition levels
#Regression
#Figure
library(Rmisc)
```

```
library(ggplot2)
library (reshape2)
library(ggsci)
setwd("D:/Submission/8-Fertilization-EMF/NC-revision/Final/data")
pHBD<-read.csv(file="Rawdata.csv")
pHBD1<-melt(pHBD,id.vars=c(1:6,23,48), measure.vars = 44:44,
                    variable.name = "Function", value.name = "value")
pHBD1$Treatment<-factor(pHBD$Treatment,levels=c("NP0","NP30","NP90","NP120"))
p1<-
ggplot(data=pHBD1,mapping=aes(x=Multidiversity,y=value,shape=Treatment,fill=Treatment,colour=Treat
ment))+
   geom_point(size=6,alpha=0.5)+
   scale_shape_manual(values = c(21,21,21,21))+
   scale_colour_manual(values =c("grey30","grey30","navy","tomato4"))+
   scale_fill_manual(values = c("white","grey70","blue3","red"))+
   geom_smooth(aes(group=Function,linetype=Function,color=Treatment), method="lm", size=1.5, se=F)+
   scale_linetype_manual(values=c(1,1,1,1))+
   ggpubr::stat_cor(aes(color = Treatment), r.digits = 2,p.digits = 2)+
   facet_wrap(~Treatment,scales="free",nrow = 2)+
   theme_bw()+
   theme(axis.text.x = element_text(size=12, angle = 0, hjust = 0.5),
            axis.text.y = element_text(size = 12),
            axis.title=element_text(size=14,face="bold"),
            axis.ticks.length = unit(0.1,"cm"),
            panel.grid = element_blank(),
            panel.background = element_blank(),
            panel.grid.major = element_blank(),
            panel.grid.minor = element_blank(),plot.background = element_blank())
plot(p1)


####Figure 7 Piecewise SEM
#full model
#Figure7
library(lme4)
library(piecewiseSEM)
setwd("D:/Submission/8-Fertilization-EMF/NC-revision/Final/data")
pSEM<-read.csv(file="Rawdata.csv")
pSEM_model<-psem(
   lmer(LabileC1~NP+(1|Block)+(1|Year),data=pSEM),
   lmer(MineralN1~NP+(1|Block)+(1|Year),data=pSEM),
   lmer(pH1~NP+(1|Block)+(1|Year),data=pSEM),
   lmer(Microbialdiversity~pH1+LabileC1+MineralN1+(1|Block)+(1|Year),data=pSEM),

lmer(Nematodediversity1~pH1+LabileC1+MineralN1+Microbialdiversity+(1|Block)+(1|Year),data=pSEM
),

lmer(Multifunction~pH1+LabileC1+MineralN1+Microbialdiversity+Nematodediversity1+(1|Block)+(1|Ye
ar),data=pSEM),
   data=pSEM
)
summary(pSEM_model)
plot(pSEM_model)

#final model
pSEM_model<-psem(
   lmer(LabileC1~NP+(1|Block)+(1|Year),data=pSEM),
```

```
  lmer(MineralN1~NP+(1|Block)+(1|Year),data=pSEM),
  lmer(pH1~NP+(1|Block)+(1|Year),data=pSEM),
  lmer(Microbialdiversity~pH1+(1|Block)+(1|Year),data=pSEM),
  lmer(Nematodediversity1~pH1+Microbialdiversity+(1|Block)+(1|Year),data=pSEM),
  lmer(Multifunction~Microbialdiversity+Nematodediversity1+(1|Block)+(1|Year),data=pSEM),
  NP%~~%Microbialdiversity,
  NP%~~%Nematodediversity1,
  NP%~~%Multifunction,
  data=pSEM
)
summary(pSEM_model)
plot(pSEM_model)


############################
```

2. R code for supplementary information

#Figure S1 is a conceptual model and does not include any data.

#Figure S2 Effects of nutrient enrichment on the diversity of soil organisms.

```
#Anova
library(psych)
library(agricolae)

setwd("D:/Submission/8-Fertilization-EMF/NC-revision/Final/data")
EMF<-read.csv(file="Rawdata.csv")
myaov=aov(Fungaldiversity~Treatment,data=EMF)
#"Bacterialdiversity,Fungaldiversity,Nematodediversity,BFdiversity
#,FFdiversity,PFdiversity,OMdiversity,PRdiversity"
summary(myaov)
LSD.test(myaov,"Treatment", p.adj="bonferroni", console=T)

#Figure
library(Rmisc)
library(ggplot2)
library (reshape2)

setwd("D:/Submission/8-Fertilization-EMF/NC-revision/Final/data")
EMF<-read.csv(file="Rawdata.csv")
EMF<-melt(EMF,id.vars=1:6, measure.vars = 11:18,
          variable.name = "Function", value.name = "value")
myhisto<-summarySE(EMF, measurevar="value",groupvars=c("Function","Treatment"))
myhisto$Treatment<-factor(myhisto$Treatment,levels=c("NP0","NP30","NP90","NP120"))
ggplot(myhisto, aes(x=Treatment, y=value,fill=Treatment,colour=Treatment))+
  geom_point(position=position_dodge(0.8),stat="identity",size=6, shape=21)+
  geom_errorbar(aes(ymin=value-
se,ymax=value+se),width=0.2,colour="black",position=position_dodge(0.8),size=0.8)+
  geom_jitter(data=EMF,aes(fill=Treatment),position=position_jitter(0.2),shape=21, size=3, alpha=0.2)+
  facet_wrap(~Function,scales="free",nrow = 2)+    #,nrow = 4
  theme_bw()+
  theme(panel.background = element_blank(),
        panel.grid.major = element_blank(),
        panel.grid.minor = element_blank(),plot.background = element_blank(),
        axis.text.x = element_text(size=12,colour="black", angle = 0, hjust = 0.5),
        axis.text.y = element_text(size = 10)) +
  scale_colour_manual(values=c("grey30","grey30","navy","tomato4"))+
  scale_fill_manual(values=c("white","grey70","blue3","red"))
```

```
#Figure S3 Effects of nutrient enrichment on soil multidiversity index.
#Anova
setwd("D:/Submission/8-Fertilization-EMF/NC-revision/Final/data")
EMF<-read.csv(file="Rawdata.csv")
myaov=aov(Multidiversity~Treatment,data=EMF)
summary(myaov)
LSD.test(myaov,"Treatment", p.adj="bonferroni", console=T)

#Figure
library(Rmisc)
library(ggplot2)
library (reshape2)

setwd("D:/Submission/8-Fertilization-EMF/NC-revision/Final/data")
EMF<-read.csv(file="Rawdata.csv")
EMF<-melt(EMF,id.vars=1:6, measure.vars = 23:23,
          variable.name = "Function", value.name = "value")
myhisto<-summarySE(EMF, measurevar="value",groupvars=c("Function","Treatment"))
myhisto$Treatment<-factor(myhisto$Treatment,levels=c("NP0","NP30","NP90","NP120"))
ggplot(myhisto, aes(x=Treatment, y=value,fill=Treatment,colour=Treatment))+
  geom_point(position=position_dodge(0.8),stat="identity",size=6, shape=21)+
  geom_errorbar(aes(ymin=value-
se,ymax=value+se),width=0.2,colour="black",position=position_dodge(0.8),size=0.8)+
  geom_jitter(data=EMF,aes(fill=Treatment),position=position_jitter(0.2),shape=21, size=3, alpha=0.2)+
  facet_wrap(~Function,scales="free",nrow = 2)+    #,nrow = 4
  theme_bw()+
  theme(panel.background = element_blank(),
        panel.grid.major = element_blank(),
        panel.grid.minor = element_blank(),plot.background = element_blank(),
        axis.text.x = element_text(size=12,colour="black", angle = 0, hjust = 0.5),
        axis.text.y = element_text(size = 10)) +
  scale_colour_manual(values=c("grey30","grey30","navy","tomato4"))+
  scale_fill_manual(values=c("white","grey70","blue3","red"))
```

```
#Figure S4 Effects of nutrient enrichment on soil individual functions.
#Anova
setwd("D:/Submission/8-Fertilization-EMF/NC-revision/Final/data")
EMF<-read.csv(file="Rawdata.csv")
myaov=aov(SOCStorage~Treatment,data=EMF)
#SOCStorage,Nstorage,Sugardegradation,Chitindegradation,Lignindegradation,
#Polymerdegradation,Pmineralization,SOCdecompositionpotential,Microbialactivity,MBC,MBN,#MBP,Ag
gregatestability,Parasiteresistance
summary(myaov)
LSD.test(myaov,"Treatment", p.adj="bonferroni", console=T)

#Figure
library(Rmisc)
library(ggplot2)
library (reshape2)

setwd("D:/Submission/8-Fertilization-EMF/NC-revision/Final/data")
EMF<-read.csv(file="Rawdata.csv")
EMF<-melt(EMF,id.vars=1:6, measure.vars = 30:43,
          variable.name = "Function", value.name = "value")
myhisto<-summarySE(EMF, measurevar="value",groupvars=c("Function","Treatment"))
myhisto$Treatment<-factor(myhisto$Treatment,levels=c("NP0","NP30","NP90","NP120"))
```

```
ggplot(myhisto, aes(x=Treatment, y=value,fill=Treatment,colour=Treatment))+
  geom_point(position=position_dodge(0.8),stat="identity",size=6, shape=21)+
  geom_errorbar(aes(ymin=value-
se,ymax=value+se),width=0.2,colour="black",position=position_dodge(0.8),size=0.8)+
  geom_jitter(data=EMF,aes(fill=Treatment),position=position_jitter(0.2),shape=21, size=3, alpha=0.2)+
  facet_wrap(~Function,scales="free",nrow = 3)+     #,nrow = 4
  theme_bw()+
  theme(panel.background = element_blank(),
        panel.grid.major = element_blank(),
        panel.grid.minor = element_blank(),plot.background = element_blank(),
        axis.text.x = element_text(size=12,colour="black", angle = 0, hjust = 0.5),
        axis.text.y = element_text(size = 10)) +
  scale_colour_manual(values=c("grey30","grey30","navy","tomato4"))+
  scale_fill_manual(values=c("white","grey70","blue3","red"))
```

#Figure S5 Effects of nutrient enrichment on the value of multifunctionality indices and the relationships among EMF indices.

```
#Anova
setwd("D:/Submission/8-Fertilization-EMF/NC-revision/Final/data")
EMF<-read.csv(file="Rawdata.csv")
myaov=aov(Multifunction~Treatment,data=EMF) #"EMF3,EMF5,EMF7"
summary(myaov)
LSD.test(myaov,"Treatment", p.adj="bonferroni", console=T)

#Figure
library(Rmisc)
library(ggplot2)
library (reshape2)

setwd("D:/Submission/8-Fertilization-EMF/NC-revision/Final/data")
EMF<-read.csv(file="Rawdata.csv")
EMF<-melt(EMF,id.vars=1:6, measure.vars = 44:47,
          variable.name = "Function", value.name = "value")
myhisto<-summarySE(EMF, measurevar="value",groupvars=c("Function","Treatment"))
myhisto$Treatment<-factor(myhisto$Treatment,levels=c("NP0","NP30","NP90","NP120"))
ggplot(myhisto, aes(x=Treatment, y=value,fill=Treatment,colour=Treatment))+
  geom_point(position=position_dodge(0.8),stat="identity",size=6, shape=21)+
  geom_errorbar(aes(ymin=value-
se,ymax=value+se),width=0.2,colour="black",position=position_dodge(0.8),size=0.8)+
  geom_jitter(data=EMF,aes(fill=Treatment),position=position_jitter(0.2),shape=21, size=3, alpha=0.2)+
  facet_wrap(~Function,scales="free",nrow = 2)+     #,nrow = 4
  theme_bw()+
  theme(panel.background = element_blank(),
        panel.grid.major = element_blank(),
        panel.grid.minor = element_blank(),plot.background = element_blank(),
        axis.text.x = element_text(size=12,colour="black", angle = 0, hjust = 0.5),
        axis.text.y = element_text(size = 10)) +
  scale_colour_manual(values=c("grey30","grey30","navy","tomato4"))+
  scale_fill_manual(values=c("white","grey70","blue3","red"))

#Regression
EMF<-read.csv(file="Rawdata.csv")
x<-EMF[,c("Multifunction")]
y<-EMF[,c("EMF3")] #"EMF3, EMF5, EMF7"
cor.test(x,y,method = "pearson")
test=lm(y~x)
test
```

```
summary(test)

#Figure
EMF<-read.csv(file="Rawdata.csv")
EMF<-melt(EMF,id.vars=c(1:6,23,44,48), measure.vars = c(45,46,47),
               variable.name = "Function", value.name = "value")
EMF$Treatment<-factor(EMF$Treatment,levels=c("NP0","NP30","NP90","NP120"))
p1<-
ggplot(data=EMF,mapping=aes(x=Multifunction,y=value,shape=Treatment,fill=Treatment,colour=Treatme
nt))+
  geom_point(size=6,alpha=0.5)+
  scale_shape_manual(values = c(21,21,21,21))+
  scale_colour_manual(values =c("grey30","grey30","navy","tomato4"))+
  scale_fill_manual(values = c("white","grey70","blue3","red"))+
  geom_smooth(aes(group=All,linetype=All), color="black",method="lm", size=1.5, se=F)+
  scale_linetype_manual(values=c(1))+
  facet_wrap(~Function,scales="free",nrow = 2)+
  theme_bw()+
  theme(axis.text.x = element_text(size=12, angle = 0, hjust = 0.5),
            axis.text.y = element_text(size = 12),
            axis.title=element_text(size=14,face="bold"),
            axis.ticks.length = unit(0.1,"cm"),
            panel.grid = element_blank(),
            panel.background = element_blank(),
            panel.grid.major = element_blank(),
            panel.grid.minor = element_blank(),plot.background = element_blank())
p1
```

#Figure S6 Relationships between soil labile C and the diversity of soil biota as influenced by nutrient enrichment.

```
#Regression
EMF<-read.csv(file="Rawdata.csv")
x<-EMF[,c("LabileC")]
y<-EMF[,c("Bacterialdiversity")]
#"Bacterialdiversity,Fungaldiversity,Nematodediversity,BFdiversity,FFdiversity,PFdiversity,
#OMdiversity,PRdiversity"
cor.test(x,y,method = "pearson")
test=lm(y~x)
test
summary(test)

#Figure
EMF<-read.csv(file="Rawdata.csv")
EMF<-melt(EMF,id.vars=c(1:10,23,44,48), measure.vars = 11:18,
               variable.name = "Function", value.name = "value")
EMF$Treatment<-factor(EMF$Treatment,levels=c("NP0","NP30","NP90","NP120"))
p1<-
ggplot(data=EMF,mapping=aes(x=LabileC,y=value,shape=Treatment,fill=Treatment,colour=Treatment))+
  geom_point(size=6,alpha=0.5)+
  scale_shape_manual(values = c(21,21,21,21))+
  scale_colour_manual(values =c("grey30","grey30","navy","tomato4"))+
  scale_fill_manual(values = c("white","grey70","blue3","red"))+
  geom_smooth(aes(group=All,linetype=All), color="black",method="lm", size=1.5, se=F)+
  scale_linetype_manual(values=c(1))+
  facet_wrap(~Function,scales="free",nrow = 2)+
  theme_bw()+
  theme(axis.text.x = element_text(size=12, angle = 0, hjust = 0.5),
```

```
            axis.text.y = element_text(size = 12),
            axis.title=element_text(size=14,face="bold"),
            axis.ticks.length = unit(0.1,"cm"),
            panel.grid = element_blank(),
            panel.background = element_blank(),
            panel.grid.major = element_blank(),
            panel.grid.minor = element_blank(),plot.background = element_blank())
p1
```

#Figure S7 Relationships between soil mineral N and the diversity of soil biota as influenced by nutrient enrichment.

```
#Regression
EMF<-read.csv(file="Rawdata.csv")
x<-EMF[,c("MineralN")]
y<-EMF[,c("Bacterialdiversity")]
#"Bacterialdiversity,Fungaldiversity,Nematodediversity,BFdiversity,FFdiversity,PFdiversity,
#OMdiversity,PRdiversity"
cor.test(x,y,method = "pearson")
test=lm(y~x)
test
summary(test)

#Figure
EMF<-read.csv(file="Rawdata.csv")
EMF<-melt(EMF,id.vars=c(1:10,23,44,48), measure.vars = 11:18,
          variable.name = "Function", value.name = "value")
EMF$Treatment<-factor(EMF$Treatment,levels=c("NP0","NP30","NP90","NP120"))
p1<-
ggplot(data=EMF,mapping=aes(x=MineralN,y=value,shape=Treatment,fill=Treatment,colour=Treatment))
+
  geom_point(size=6,alpha=0.5)+
  scale_shape_manual(values = c(21,21,21,21))+
  scale_colour_manual(values =c("grey30","grey30","navy","tomato4"))+
  scale_fill_manual(values = c("white","grey70","blue3","red"))+
  geom_smooth(aes(group=All,linetype=All), color="black",method="lm", size=1.5, se=F)+
  scale_linetype_manual(values=c(1))+
  facet_wrap(~Function,scales="free",nrow = 2)+
  theme_bw()+
  theme(axis.text.x = element_text(size=12, angle = 0, hjust = 0.5),
            axis.text.y = element_text(size = 12),
            axis.title=element_text(size=14,face="bold"),
            axis.ticks.length = unit(0.1,"cm"),
            panel.grid = element_blank(),
            panel.background = element_blank(),
            panel.grid.major = element_blank(),
            panel.grid.minor = element_blank(),plot.background = element_blank())
p1
```

#Figure S8 Relationships between soil available P and the diversity of soil biota as influenced by nutrient enrichment.

```
#Regression
EMF<-read.csv(file="Rawdata.csv")
x<-EMF[,c("AvailableP")]
y<-EMF[,c("Bacterialdiversity")]
#"Bacterialdiversity,Fungaldiversity,Nematodediversity,BFdiversity,FFdiversity,PFdiversity,
#OMdiversity,PRdiversity"
```

```
cor.test(x,y,method = "pearson")
test=lm(y~x)
test
summary(test)

#Figure
EMF<-read.csv(file="Rawdata.csv")
EMF<-melt(EMF,id.vars=c(1:10,23,44,48), measure.vars = 11:18,
                variable.name = "Function", value.name = "value")
EMF$Treatment<-factor(EMF$Treatment,levels=c("NP0","NP30","NP90","NP120"))
p1<-
ggplot(data=EMF,mapping=aes(x=AvailableP,y=value,shape=Treatment,fill=Treatment,colour=Treatment))
+
  geom_point(size=6,alpha=0.5)+
  scale_shape_manual(values = c(21,21,21,21))+
  scale_colour_manual(values =c("grey30","grey30","navy","tomato4"))+
  scale_fill_manual(values = c("white","grey70","blue3","red"))+
  geom_smooth(aes(group=All,linetype=All), color="black",method="lm", size=1.5, se=F)+
  scale_linetype_manual(values=c(1))+
  facet_wrap(~Function,scales="free",nrow = 2)+
  theme_bw()+
  theme(axis.text.x = element_text(size=12, angle = 0, hjust = 0.5),
          axis.text.y = element_text(size = 12),
          axis.title=element_text(size=14,face="bold"),
          axis.ticks.length = unit(0.1,"cm"),
          panel.grid = element_blank(),
          panel.background = element_blank(),
          panel.grid.major = element_blank(),
          panel.grid.minor = element_blank(),plot.background = element_blank())
p1
```

#Figure S9 Relationships between soil pH and the diversity of soil biota as influenced by nutrient enrichment.

```
#Regression
EMF<-read.csv(file="Rawdata.csv")
x<-EMF[,c("pH")]
y<-EMF[,c("Bacterialdiversity")]
#"Bacterialdiversity,Fungaldiversity,Nematodediversity,BFdiversity,FFdiversity,PFdiversity,
#OMdiversity,PRdiversity"
cor.test(x,y,method = "pearson")
test=lm(y~x)
test
summary(test)

#Figure
EMF<-read.csv(file="Rawdata.csv")
EMF<-melt(EMF,id.vars=c(1:10,23,44,48), measure.vars = 11:18,
                variable.name = "Function", value.name = "value")
EMF$Treatment<-factor(EMF$Treatment,levels=c("NP0","NP30","NP90","NP120"))
p1<-ggplot(data=EMF,mapping=aes(x=pH,y=value,shape=Treatment,fill=Treatment,colour=Treatment))+
  geom_point(size=6,alpha=0.5)+
  scale_shape_manual(values = c(21,21,21,21))+
  scale_colour_manual(values =c("grey30","grey30","navy","tomato4"))+
  scale_fill_manual(values = c("white","grey70","blue3","red"))+
  geom_smooth(aes(group=All,linetype=All), color="black",method="lm", size=1.5, se=F)+
  scale_linetype_manual(values=c(1))+
  facet_wrap(~Function,scales="free",nrow = 2)+
```

```
  theme_bw()+
  theme(axis.text.x = element_text(size=12, angle = 0, hjust = 0.5),
        axis.text.y = element_text(size = 12),
        axis.title=element_text(size=14,face="bold"),
        axis.ticks.length = unit(0.1,"cm"),
        panel.grid = element_blank(),
        panel.background = element_blank(),
        panel.grid.major = element_blank(),
        panel.grid.minor = element_blank(),plot.background = element_blank())
p1
```

#Figure S10 Relationships between soil labile C and individual soil functions as influenced by nutrient enrichment.

```
#Regression
EMF<-read.csv(file="Rawdata.csv")
x<-EMF[,c("LabileC")]
y<-EMF[,c("SOCStorage")]
#SOCStorage,Nstorage,Sugardegradation,Chitindegradation,Lignindegradation,
#Polymerdegradation,Pmineralization,SOCdecompositionpotential,Microbialactivity,MBC,MBN,#MBP,Aggregatestability,Parasiteresistance
cor.test(x,y,method = "pearson")
test=lm(y~x)
test
summary(test)

#Figure
EMF<-read.csv(file="Rawdata.csv")
EMF<-melt(EMF,id.vars=c(1:10,23,44,48), measure.vars = 30:43,
          variable.name = "Function", value.name = "value")
EMF$Treatment<-factor(EMF$Treatment,levels=c("NP0","NP30","NP90","NP120"))
p1<-
ggplot(data=EMF,mapping=aes(x=LabileC,y=value,shape=Treatment,fill=Treatment,colour=Treatment))+
  geom_point(size=6,alpha=0.5)+
  scale_shape_manual(values = c(21,21,21,21))+
  scale_colour_manual(values =c("grey30","grey30","navy","tomato4"))+
  scale_fill_manual(values = c("white","grey70","blue3","red"))+
  geom_smooth(aes(group=All,linetype=All), color="black",method="lm", size=1.5, se=F)+
  scale_linetype_manual(values=c(1))+
  facet_wrap(~Function,scales="free",nrow = 3)+
  theme_bw()+
  theme(axis.text.x = element_text(size=12, angle = 0, hjust = 0.5),
        axis.text.y = element_text(size = 12),
        axis.title=element_text(size=14,face="bold"),
        axis.ticks.length = unit(0.1,"cm"),
        panel.grid = element_blank(),
        panel.background = element_blank(),
        panel.grid.major = element_blank(),
        panel.grid.minor = element_blank(),plot.background = element_blank())
p1
```

#Figure S11 Relationships between soil mineral N and individual soil functions as influenced by nutrient enrichment.

```
#Regression
EMF<-read.csv(file="Rawdata.csv")
x<-EMF[,c("MineralN")]
y<-EMF[,c("SOCStorage")]
#SOCStorage,Nstorage,Sugardegradation,Chitindegradation,Lignindegradation,
```

```
#Polymerdegradation,Pmineralization,SOCdecompositionpotential,Microbialactivity,MBC,MBN,#MBP,Aggregatestability,Parasiteresistance
cor.test(x,y,method = "pearson")
test=lm(y~x)
test
summary(test)

#Figure
EMF<-read.csv(file="Rawdata.csv")
EMF<-melt(EMF,id.vars=c(1:10,23,44,48), measure.vars = 30:43,
               variable.name = "Function", value.name = "value")
EMF$Treatment<-factor(EMF$Treatment,levels=c("NP0","NP30","NP90","NP120"))
p1<-
ggplot(data=EMF,mapping=aes(x=MineralN,y=value,shape=Treatment,fill=Treatment,colour=Treatment))
+
  geom_point(size=6,alpha=0.5)+
  scale_shape_manual(values = c(21,21,21,21))+
  scale_colour_manual(values =c("grey30","grey30","navy","tomato4"))+
  scale_fill_manual(values = c("white","grey70","blue3","red"))+
  geom_smooth(aes(group=All,linetype=All), color="black",method="lm", size=1.5, se=F)+
  scale_linetype_manual(values=c(1))+
  facet_wrap(~Function,scales="free",nrow = 3)+
  theme_bw()+
  theme(axis.text.x = element_text(size=12, angle = 0, hjust = 0.5),
          axis.text.y = element_text(size = 12),
          axis.title=element_text(size=14,face="bold"),
          axis.ticks.length = unit(0.1,"cm"),
          panel.grid = element_blank(),
          panel.background = element_blank(),
          panel.grid.major = element_blank(),
          panel.grid.minor = element_blank(),plot.background = element_blank())
p1
```

#Figure S12 Relationships between soil available P and individual soil functions as influenced by nutrient enrichment.

```
#Regression
EMF<-read.csv(file="Rawdata.csv")
x<-EMF[,c("AvailableP")]
y<-EMF[,c("SOCStorage")]
#SOCStorage,Nstorage,Sugardegradation,Chitindegradation,Lignindegradation,
#Polymerdegradation,Pmineralization,SOCdecompositionpotential,Microbialactivity,MBC,MBN,#MBP,Aggregatestability,Parasiteresistance
cor.test(x,y,method = "pearson")
test=lm(y~x)
test
summary(test)

#Figure
EMF<-read.csv(file="Rawdata.csv")
EMF<-melt(EMF,id.vars=c(1:10,23,44,48), measure.vars = 30:43,
               variable.name = "Function", value.name = "value")
EMF$Treatment<-factor(EMF$Treatment,levels=c("NP0","NP30","NP90","NP120"))
p1<-
ggplot(data=EMF,mapping=aes(x=AvailableP,y=value,shape=Treatment,fill=Treatment,colour=Treatment))
+
  geom_point(size=6,alpha=0.5)+
```

```
  scale_shape_manual(values = c(21,21,21,21))+
  scale_colour_manual(values =c("grey30","grey30","navy","tomato4"))+
  scale_fill_manual(values = c("white","grey70","blue3","red"))+
  geom_smooth(aes(group=All,linetype=All), color="black",method="lm", size=1.5, se=F)+
  scale_linetype_manual(values=c(1))+
  facet_wrap(~Function,scales="free",nrow = 3)+
  theme_bw()+
  theme(axis.text.x = element_text(size=12, angle = 0, hjust = 0.5),
        axis.text.y = element_text(size = 12),
        axis.title=element_text(size=14,face="bold"),
        axis.ticks.length = unit(0.1,"cm"),
        panel.grid = element_blank(),
        panel.background = element_blank(),
        panel.grid.major = element_blank(),
        panel.grid.minor = element_blank(),plot.background = element_blank())
p1
```

#Figure S13 Relationships between soil pH and individual soil functions as influenced by nutrient enrichment.

```
#Regression
EMF<-read.csv(file="Rawdata.csv")
x<-EMF[,c("pH")]
y<-EMF[,c("SOCStorage")]
#SOCStorage,Nstorage,Sugardegradation,Chitindegradation,Lignindegradation,
#Polymerdegradation,Pmineralization,SOCdecompositionpotential,Microbialactivity,MBC,MBN,#MBP,Aggregatestability,Parasiteresistance
cor.test(x,y,method = "pearson")
test=lm(y~x)
test
summary(test)

#Figure
EMF<-read.csv(file="Rawdata.csv")
EMF<-melt(EMF,id.vars=c(1:10,23,44,48), measure.vars = 30:43,
          variable.name = "Function", value.name = "value")
EMF$Treatment<-factor(EMF$Treatment,levels=c("NP0","NP30","NP90","NP120"))
p1<-ggplot(data=EMF,mapping=aes(x=pH,y=value,shape=Treatment,fill=Treatment,colour=Treatment))+
  geom_point(size=6,alpha=0.5)+
  scale_shape_manual(values = c(21,21,21,21))+
  scale_colour_manual(values =c("grey30","grey30","navy","tomato4"))+
  scale_fill_manual(values = c("white","grey70","blue3","red"))+
  geom_smooth(aes(group=All,linetype=All), color="black",method="lm", size=1.5, se=F)+
  scale_linetype_manual(values=c(1))+
  facet_wrap(~Function,scales="free",nrow = 3)+
  theme_bw()+
  theme(axis.text.x = element_text(size=12, angle = 0, hjust = 0.5),
        axis.text.y = element_text(size = 12),
        axis.title=element_text(size=14,face="bold"),
        axis.ticks.length = unit(0.1,"cm"),
        panel.grid = element_blank(),
        panel.background = element_blank(),
        panel.grid.major = element_blank(),
        panel.grid.minor = element_blank(),plot.background = element_blank())
p1
```

#Figure S14 Nutrient enrichment weakened the relationship between the diversity of single soil biota

group and EMF at all three nutrient addition levels.

```
#Regression
EMF<-read.csv(file="Rawdata.csv")
EMF<-EMF[EMF$Treatment=="NP0",] #"NP0,NP30,NP90,NP120"
x<-EMF[,c("Nematodediversity1")] ##"Bacterialdiversity1,Fungaldiversity1,Nematodediversity1"
y<-EMF[,c("Multifunction")]
cor.test(x,y,method = "pearson")
test=lm(y~x)
test
summary(test)

library(Rmisc)
library(ggplot2)
library (reshape2)
library(ggsci)
EMF<-read.csv(file="Rawdata.csv")
EMF1<-melt(EMF,id.vars=c(1:10,23,44,48), measure.vars = 54:56,
                 variable.name = "Function", value.name = "value")
EMF1$Treatment<-factor(EMF$Treatment,levels=c("NP0","NP30","NP90","NP120"))
p1<-
ggplot(data=EMF1,mapping=aes(x=value,y=Multifunction,color=Treatment,fill=Treatment,shape=Functio
n))+
  geom_point(aes(color=Treatment),size=3,alpha=0.5)+
  scale_shape_manual(values = c(21,22,24))+
  scale_colour_manual(values =c("grey30","grey30","navy","tomato4"))+
  scale_fill_manual(values = c("white","grey70","blue3","red"))+
  geom_smooth(aes(group=Function,linetype=Function),color="black", method="lm", size=1.5, se=F)+
  scale_linetype_manual(values=c(1,2,3))+
  ggpubr::stat_cor(aes(color = Treatment), r.digits = 2,p.digits = 2)+
  facet_wrap(~Treatment,scales="free",nrow = 2)+
  theme_bw()+
  theme(axis.text.x = element_text(size=12,colour="black", angle = 0, hjust = 0.5),
        axis.text.y = element_text(size = 12,colour="black"),
        axis.title=element_text(size=14,face="bold"),
        axis.ticks.length = unit(0.1,"cm"),
        #axis.ticks=element_line(linetype=1,color="black",size=1),
        #panel.spacing.x = unit(10, 'mm'),
        legend.position="right",
        panel.grid = element_blank(),
        panel.background = element_blank(),
        panel.grid.major = element_blank(),
        panel.grid.minor = element_blank(),plot.background = element_blank())
plot(p1)
```

#Figure S15 Effects of nutrient enrichment on microbial community structure

```
#Anova
EMF<-read.csv(file="Rawdata.csv")
myaov=aov(Bacterialbiomass~Treatment,data=EMF)
#"Bacterialbiomass, Fungalbiomass, BaFu, GP,GN,GPGN"
summary(myaov)
LSD.test(myaov,"Treatment", p.adj="bonferroni", console=T)


#Figure
setwd("D:/Submission/8-Fertilization-EMF/NC-revision/Final/data")
EMF<-read.csv(file="Rawdata.csv")
EMF<-melt(EMF,id.vars=1:6, measure.vars = 24:29,
```

```
                   variable.name = "Function", value.name = "value")
myhisto<-summarySE(EMF, measurevar="value",groupvars=c("Function","Treatment"))
myhisto$Treatment<-factor(myhisto$Treatment,levels=c("NP0","NP30","NP90","NP120"))
ggplot(myhisto, aes(x=Treatment, y=value,fill=Treatment,colour=Treatment))+
    geom_point(position=position_dodge(0.8),stat="identity",size=6, shape=21)+
    geom_errorbar(aes(ymin=value-
se,ymax=value+se),width=0.2,colour="black",position=position_dodge(0.8),size=0.8)+
    geom_jitter(data=EMF,aes(fill=Treatment),position=position_jitter(0.2),shape=21, size=3, alpha=0.2)+
    facet_wrap(~Function,scales="free",nrow = 2)+      #,nrow = 4
    theme_bw()+
    theme(panel.background = element_blank(),
              panel.grid.major = element_blank(),
              panel.grid.minor = element_blank(),plot.background = element_blank(),
              axis.text.x = element_text(size=12,colour="black", angle = 0, hjust = 0.5),
              axis.text.y = element_text(size = 10)) +
    scale_colour_manual(values=c("grey30","grey30","navy","tomato4"))+
    scale_fill_manual(values=c("white","grey70","blue3","red"))
```

#Figure S16 Effects of nutrient enrichment on the relative abundance of nematode genera.

```
library(ggplot2)
library(Rmisc)
library (reshape2)
library(BBmisc)
library(readxl)

spe <- read_excel("D:/Submission/8-Fertilization-EMF/NC-revision/Final/data/Soil biota community.xlsx",
sheet = 1)

#spe<-normalize(spe, method="range", margin=2)
spe1 <- melt(spe,id.vars=1:6,measurevar=7:82,variable.name = "nematode", value.name = "value")
#need to insert a column regarding treatment
dfc <- summarySE(spe1, measurevar="value",groupvars=c("nematode","Treatment"))

dfc$Treatment <- factor(dfc$Treatment, levels =unique(spe$Treatment))
#write.csv(dfc,file="dfc.csv")

p <- ggplot(dfc, aes(x=Treatment,y=nematode)) +
    xlab("samples") +
    theme_classic() +
    theme(axis.ticks = element_blank(),
              axis.line = element_blank()) +
    labs(x="Site",y="Nematode genera")+
    theme(panel.grid.major = element_blank()) +
    theme(legend.key=element_blank())    +
    theme(axis.text.x=element_text(size=12,colour="black",angle=0,hjust=0.5, vjust=1),
              axis.text.y = element_text(size = 11,colour="black"),) +
    theme(legend.position="right") +
    geom_tile(aes(fill=value)) +
    scale_fill_gradient2("Relative abundance",
                                         low = "navy",
                                         high = "red3")
p###9.4*6.25
ggsave("Relative abundance.pdf", plot=p, width = 150, height = 550, units = "mm")
```

Supplementary references

1. Bossio, D. A., Scow, K. M., Gunapala, N. & Graham, K. J. Determinants of soil microbial communities: Effects of agricultural management, season, and soil type on phospholipid fatty acid profiles. *Microb. Ecol.* **36**, 1–12 (1998).
2. Delgado-Baquerizo, M. *et al.* Multiple elements of soil biodiversity drive ecosystem functions across biomes. *Nat. Ecol. Evol.* **4**, 210–220 (2020).
3. Jiao, S., Lu, Y. & Wei, G. Soil multitrophic network complexity enhances the link between biodiversity and multifunctionality in agricultural systems. *Glob. Chang. Biol.* **28**, 140–153 (2022).
4. Baldock, J. A. & Smernik, R. J. Chemical composition and bioavailability of thermally altered Pinus resinosa (Red pine) wood. *Org. Geochem.* **33**, 1093–1109 (2002).
5. Fanin, N. *et al.* Consistent effects of biodiversity loss on multifunctionality across contrasting ecosystems. *Nat. Ecol. Evol.* **2**, 269–278 (2018).
6. Fierer, N. & Schimel, J. P. A Proposed Mechanism for the Pulse in Carbon Dioxide Production Commonly Observed Following the Rapid Rewetting of a Dry Soil. *Soil Sci. Soc. Am. J.* **67**, 798–805 (2003).
7. García, C., Hernández, T. & Costa, F. Microbial activity in soils under mediterranean environmental conditions. *Soil Biol. Biochem.* **26**, 1185–1191 (1994).
8. Liu, T. *et al.* Earthworms Coordinate Soil Biota to Improve Multiple Ecosystem Functions. *Curr. Biol.* **29**, 3420-3429.e5 (2019).
9. Amézketa, E. Soil aggregate stability: A review. *J. Sustain. Agric.* **14**, 83–151 (1999).
10. Bai, T., Wang, P., Ye, C. & Hu, S. Form of nitrogen input dominates N effects on root growth and soil aggregation: A meta-analysis. *Soil Biol. Biochem.* **157**, 108251 (2021).
111. Bakonyi, G. & Nagy, P. Temperature- and moisture-induced changes in the structure of the nematode fauna of a semiarid grassland - patterns and mechanisms. *Glob. Chang. Biol.* **6**, 697–707 (2000).
12. Hu, Z. *et al.* Responses of rice paddy micro-food webs to elevated CO_2 are modulated by nitrogen fertilization and crop cultivars. *Soil Biol. Biochem.* **114**, 104–113 (2017).
13. Topalović, O. & Geisen, S. Nematodes as suppressors and facilitators of plant performance. *New Phytol.* (2023).
